# Supplementary material for: Inflammatory factors and risk of lung adenocarcinoma: a Mendelian randomization study mediated by blood metabolites
Source: Front Endocrinol (Lausanne). 2024 Aug 27;15:1446863. doi: 10.3389/fendo.2024.1446863 (PMC11384989; doi:10.3389/fendo.2024.1446863)
Supplement: Supplementary file 1 [file DataSheet1.zip › supplementary files/S2/GCST90200992/forest.pdf]

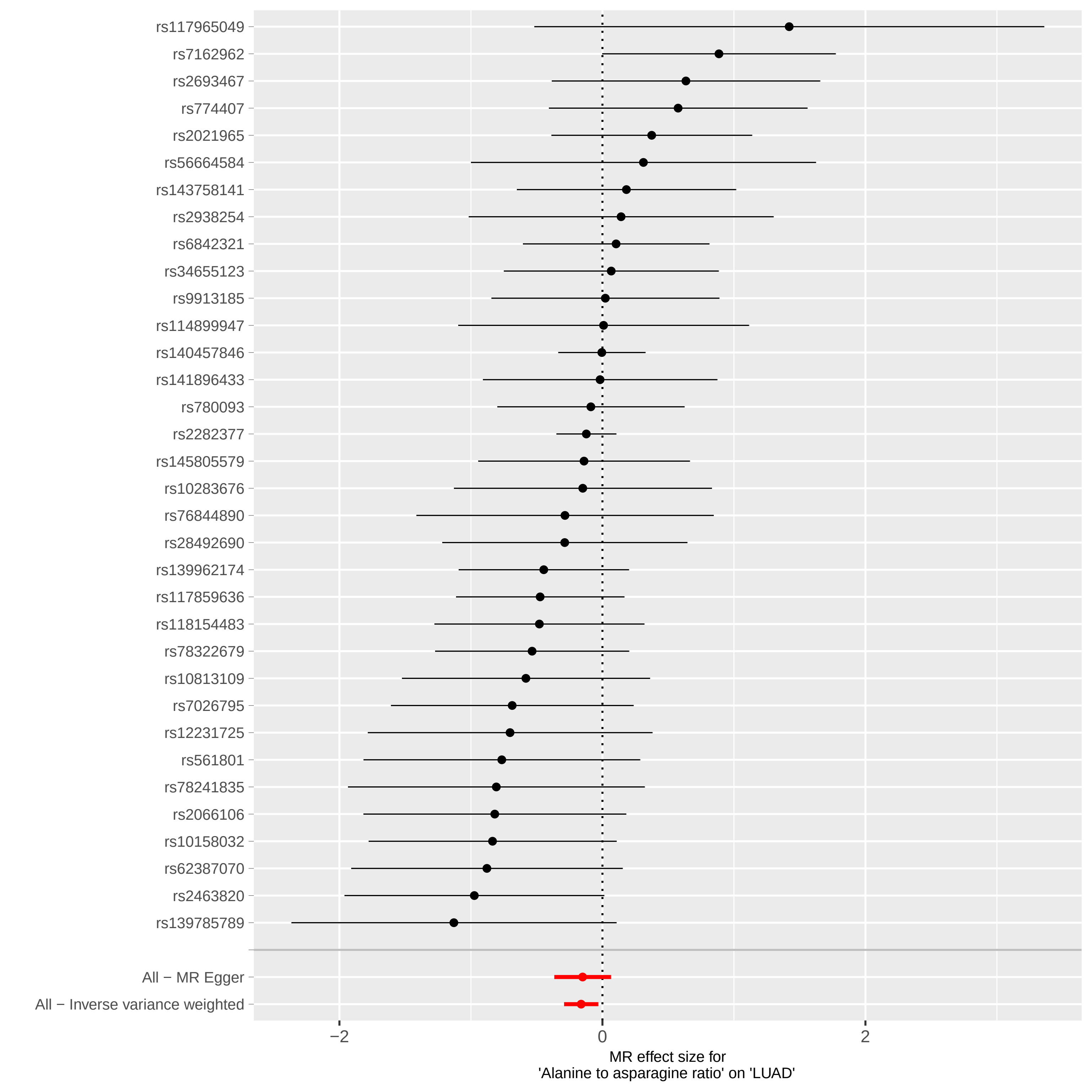
rs117965049
rs7162962
rs2693467
rs774407
rs2021965
rs56664584
rs143758141
rs2938254
rs6842321
rs34655123
rs9913185
rs114899947
rs140457846
rs141896433
rs780093
rs2282377
rs145805579
rs10283676
rs76844890
rs28492690
rs139962174
rs117859636
rs118154483
rs78322679
rs10813109
rs7026795
rs12231725
rs561801
rs78241835
rs2066106
rs10158032
rs62387070
rs2463820
rs139785789
All − MR Egger
All − Inverse variance weighted
−2
0
2
MR effect size for
'Alanine to asparagine ratio' on 'LUAD'
